# Diagnostic Challenges of Sinonasal Pleomorphic Adenoma

**DOI:** 10.7759/cureus.54010

**Published:** 2024-02-11

**Authors:** Stefan Konsulov, Denis Milkov, Daniel Markov, Elena G Poryazova

**Affiliations:** 1 Department of Otorhinolaryngology, Medical University of Plovdiv, Plovdiv, BGR; 2 Department of Otorhinolaryngology, University Hospital Kaspela, Plovdiv, BGR; 3 Department of General and Clinical Pathology, Medical University of Plovdiv, Plovdiv, BGR; 4 Department of Clinical Pathology, University Hospital Pulmed, Plovdiv, BGR

**Keywords:** sinonasal tumor, epiphora, unilateral nasal obstruction, nasal deformity, pleomorphic adenoma

## Abstract

Pleomorphic adenomas (PAs) are benign tumors of the salivary glands. Rarely, they arise in the sinonasal cavity, presenting as well-defined, homogeneous soft tissue masses, causing expansive bony changes. The significance of PAs is the possibility of giving rise to malignant carcinoma - “carcinoma ex-pleomorphic adenoma” (CXPA).Here, we present the case of a 64-year-old female complaining of progressive unilateral congestion and external nose deformation, mostly along the left contour of the radix, with epiphora of the ipsilateral eye. Eventually, a tumor began protruding from the left naris. The computed tomography excluded osteolysis, while the surgical procedure discovered the inferior turbinate as the origin of the tumor. In addition, the ipsilateral maxillary sinus was found to have developed secondary sinusitis. After complete surgical excision, the histological result was sinonasal melanoma, but following no progression of the disease, a second pathologist with additional immunohistochemical markers (HMB-45 (human melanoma black 45) negative, Melan-A (melanoma antigen recognized by T-cells 1) negative, S100 (protein soluble in 100% ammonium sulfate at neutral pH) positive, panCK AE1/AE3 (pan cytokeratin antibodies AE1 and AE3) negative, p63 (tumor protein 63) negative, Ki-67 (marker of proliferation Kiel 67) 10%, CD68 (cluster of differentiation 68) negative, CK7 (cytokeratin 7) negative, and CDX2 (caudal-type homeobox 2) negative) placed the definitive diagnosis of PA.PA of the inferior turbinate is an extremely rare finding, with the clinical symptoms being unspecific. Sometimes, SOX-10 (SRY-box transcription factor 10) positivity can mislead to malignant melanoma, as in our case, which is why a broad panel of immunohistochemical markers is critical for the definitive diagnosis.

## Introduction

Pleomorphic adenomas (PAs) are the most common benign tumors of the salivary glands, affecting primarily women in their third to sixth decades of life [1]. The parotid and submandibular glands are affected in about 75% and 15% of cases, respectively, while only 10% of PAs arise from the minor salivary glands [2].

Intranasal PAs are rare [3,4], arising from the nasal septum in 53% of cases, lateral nasal wall and nasopharynx both in about 14% of cases, and paranasal involvement in 11.5% of cases, mostly of the maxillary sinus [1]. PAs of the inferior nasal turbinate are especially rare [5].

The etiopathogenesis of PAs remains unknown. The prevailing theories regarding their origin include vomeronasal organ residues [6] and mature salivary gland tissue [7]. Histologically, sinonasal and nasopharyngeal PAs are predominantly epithelial, with less mesenchymal stroma, and they are devoid of capsules, in comparison with mixed tumors of the major salivary glands [8].

Clinically, sinonasal PAs are usually well-defined, homogeneous soft tissue masses, causing expansive bony changes [1]. The most common symptoms of sinonasal PAs are unilateral nasal congestion and epistaxis, while additional symptoms may include mucopurulent rhinorrhea, nasal swelling, and external deformities [1].

The significance of PAs is the possibility of giving rise to malignant carcinoma, namely, “carcinoma ex-pleomorphic adenoma” (CXPA), most commonly adenocarcinoma [9]. A recent rapid growth in a long-term neoplasm, as well as headaches, visual changes, facial paraesthesias, or pain due to adjacent structure invasion, may signal the presence of CXPA. In addition, osteolysis on computed tomography is an alarming finding in the presence of malignancy [1]. Patients diagnosed with an invasive type of CXPA have a five-year survival rate of approximately 30% [10].

## Case presentation

**Figure 1:**
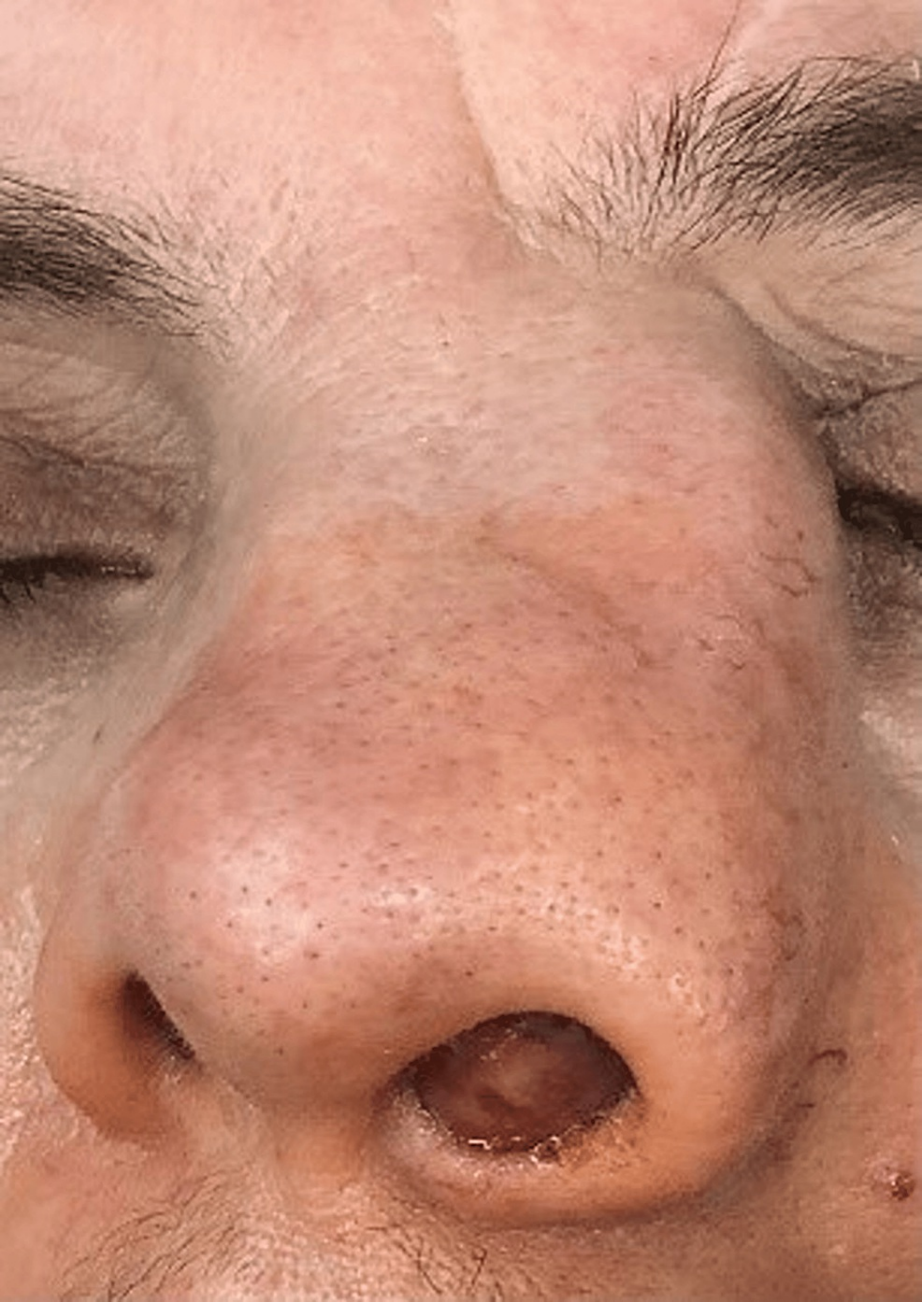
Pleomorphic adenoma of the left nasal cavity, nasal vestibule, and naris, leading to deformity of the external nose.

We present the case of a 64-year-old female complaining of progressive left-sided nasal congestion over the past five years. After being surgically treated at another center with a partial removal of a left-sided tumor with a histologic result, i.e., a sinonasal polyp, her unilateral nasal congestion persisted. In addition, about two years ago, a progressive deformation of the external nose began, mostly along the left contour of the radix, with epiphora of the ipsilateral eye. These complaints persisted over time, with a gradual development of facial deformity, completely blocked nasal breathing on the left, and prominence of a tumor from the left naris (Figure 1). The patient denied having pain, bleeding, and visual changes at any moment.

**Figure 2:**
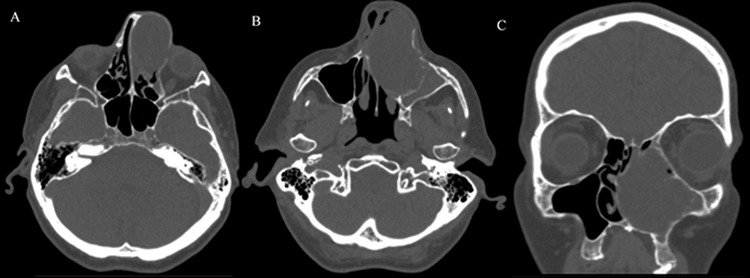
Pre-surgical computed tomography of an inferior turbinate pleomorphic adenoma and secondary maxillary sinusitis. A and B: axial view, C: coronal view

A computed tomography study described polypoid hypertrophy of the mucosa of the left maxillary sinus, with prominence through the medial wall and involvement of the left ethmoidal cells and left nasal cavity, with a density of 28 Hounsfield units (HU). No osteolysis was observed (Figure 2).

**Figure 3:**
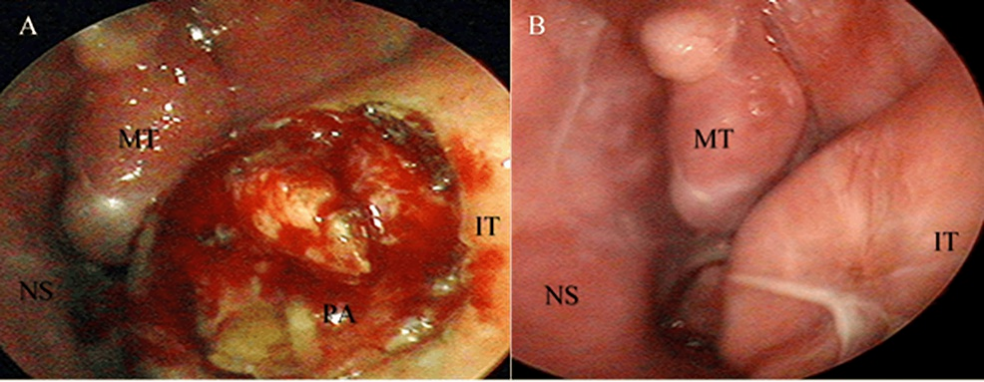
Pleomorphic adenoma of the inferior turbinate. A: surgery, B: six months post-operative. PA: pleomorphic adenoma, NS: nasal septum, MT: middle turbinate, IT: inferior turbinate

The surgical procedure began with a partial removal of the tumor, after which its origin was found to be the inferior turbinate, leading to its destruction (Figure 3A). Intraoperative frozen section histology found edematous mucosa, most likely polypoid. Then, the remainder of the tumor was removed, all the way to the posterior end of the inferior turbinate, and electrocautery was performed at the site of origin on the inferior turbinate. After performing maxillary antrostomy, the maxillary sinus was found to have developed secondary sinusitis.

The postoperative histological report described an abundant fragmented material from a hyperplastic polypoid nasal formation, lined with normal nasal epithelium and edematous stroma, among which, along with normal glands, there are single and clustered relatively monomorphic "balloon-shaped" cells, which are SOX-10 (SRY-box transcription factor 10) positive, Ki-67 (marker of proliferation Kiel 67) about 20% and CD68 (cluster of differentiation 68), CDX2 (caudal-type homeobox 2), and CK7 (cytokeratin 7) are negative. The histological pattern and positive expression of SOX-10 were suggestive of a primary mucosal sinonasal melanoma.

This histologic result led us to refer the patient for positron emission tomography due to the aggressive nature of sinonasal melanomas, only to incidentally discover an intramammary metabolic lymph node in the right mammary gland. Subsequently, she was diagnosed and treated for invasive ductal carcinoma of the superomedial quadrant of the right mammary gland cT1 N0 M0.

**Figure 4:**
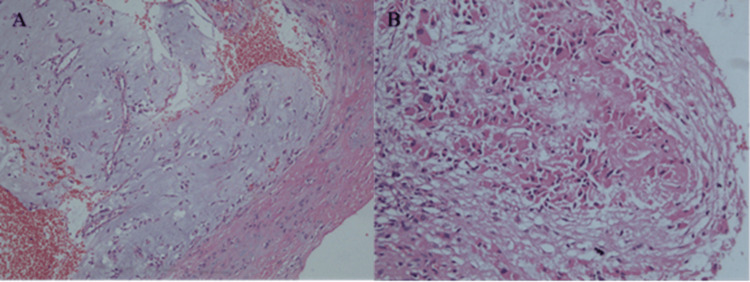
Fragmented sinonasal biopsy of pleomorphic adenoma, hematoxylin-eosin staining, magnification x100. A: Epithelial and myoepithelial cells, forming acinar and trabecular structures, zones with chondroid and myxoid stroma with single stellate cells. B: Small cells with eosinophilic cytoplasm and an eccentric oval nucleus without nucleoli, with a rhabdoid morphology.

There was no progression of the disease at the follow-up endoscopy six months post-operative, which raised the question as to whether our primary histological result was accurate (Figure 3B). This led us to review the tissue from the surgery by a second pathologist, who described epithelial and myoepithelial cells, forming acinar and trabecular structures, zones with chondroid and myxoid stroma with single stellate cells (Figure 4A). Moreover, small cells with eosinophilic cytoplasm and an eccentric oval nucleus without nucleoli, with a rhabdoid morphology, were described (Figure 4B). The margins were clear of disease.

**Figure 5:**
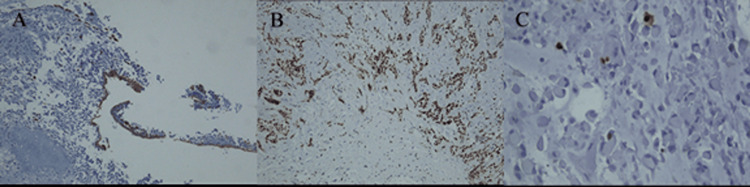
Fragmented sinonasal biopsy of pleomorphic adenoma: immunohistochemistry study. A: Focal positivity for CK, magnification x100. B) positivity for SOX-10, magnification x100. C) Low proliferative index with Ki-67 less than 10%, magnification x200.

The immunohistochemistry result was focal positivity for CK (Figure 5A), SOX-10 positivity (Figure 5B), and a low proliferative index with Ki-67 less than 10% (Figure 5C), suggestive of PA. Additional immunohistochemistry melanocyte markers, as well as markers for epithelial and myoepithelial differentiation, were studied, which excluded malignant melanoma and led to the definitive diagnosis of PA (Table 1).

**Table 1:** Immunohistochemical markers of pleomorphic adenoma.

НМВ-45	Melan-A	S100	panCK AE1/АЕ3	р63	Ki-67	CD68	СК7	CDX2
Negative	Negative	Positive	Negative	Negative	10%	Negative	Negative	Negative

Concomitant diseases were non-insulin-dependent diabetes mellitus, cholecystectomy, and thyroidectomy. The postoperative treatment included antibiotics and intranasal saline spray. The follow-up biopsy at 12 months post-surgically found no presence of disease.

## Discussion

Sinonasal PAs have been found to originate from the nasal septum, more commonly in females, with the mean age at diagnosis being 45 years [3,6]. The patient that we presented was also a female, but she was diagnosed at the age of 64 years and the tumor was found to originate from the inferior turbinate - a particularly rare finding. Only Erol et al. described a similar case of a 62-year-old female with PA of the inferior turbinate, presenting with a four-year duration of unilateral nasal obstruction and epiphora [11]. Our patient did not experience epiphora but, instead, suffered from significant external nasal deformity. According to the series and literature review of Rha et al., other symptoms that may be observed in sinonasal PAs include epistaxis and mucopurulent rhinorrhea [3], which our patient did not have. A shortcoming of our case presentation is the use of only a single imaging modality. Retrospectively, we believe that an expanded imaging protocol, including magnetic resonance imaging and ultrasound, all with contrast, would have enhanced our preoperative diagnosis and treatment planning, as well as follow-up [12].

The rarity of intranasal PAs and their complex histology make the diagnosis difficult. Generally, they consist of a mixture of epithelial and mesenchymal areas. The epithelial component includes multiple cell types - cuboidal, basaloid, squamous, sebaceous, and clear cells - forming epithelial sheets or duct-like structures. The ducts are usually small, contain eosinophilic secretory material, and have cuboidal luminal cells and a layer of myoepithelial cells. The mesenchymal component is chondromyxoid, with the inner ductal cells expressing pancytokeratin, while the neoplastic myoepithelial cells expressing pancytokeratin, vimentin, and S-100 protein [11].

An important contribution of the presentation of the current case is that sinonasal PA may be histologically misdiagnosed. Concerning the expression of SOX-10, it is not definitive and unique for the diagnosis of malignant melanoma. It occurs in most cases of PA and adenocarcinomas of the salivary glands (except ductal ones and those with oncocytic differentiation). The histological diagnosis of PAs can be confirmed by immunohistochemical staining for positive expression of factors, such as cytokeratins, vimentin, S100, SMA, and glial fibrillary acidic protein (GFAP) [13]. In our patient, we studied the markers HMB-45 (human melanoma black 45), Melan-A (melanoma antigen recognized by T-cells 1), S100 (protein soluble in 100% ammonium sulfate at neutral pH), panCK AE1/AE3 (pan cytokeratin antibodies AE1 and AE3), p63 (tumor protein 63), Ki-67 (marker of proliferation Kiel 67), CD68 (cluster of differentiation 68), CK7, and CDX2. Importantly, the S100 positivity in our patient necessitates a differential diagnosis of nasopharyngeal carcinoma with additional immunohistochemical markers [14].

Considering the presence of CXPA in about 8% of patients with a PA, it must be actively sought [3]. The proliferation marker Ki-67, as well as human epidermal growth factor receptor 2 (HER-2), and overexpression of TP53 genes and proteins by molecular genetic analysis may serve in the identification of CXPA [1,15]. In our study, the Ki-67 was less than 10%.

The treatment of choice for sinonasal PA is complete surgical excision with clear resection margins [16]. Paranasal sinus PAs are associated with a recurrence of 10%, over a follow-up of two years. Rha et al. additionally reported from their literature review and experienced a higher rate of recurrence of patients treated by piece-meal resection, as compared to en bloc resection [3]. Due to the size of the PA in our case, we were not able to perform en-bloc resection, needing to de-bulk the tumor, before discovering its site of origin, but our follow-up biopsies over one year revealed that the patient is disease-free.

## Conclusions

PA of the inferior turbinate is an extremely rare finding, with the clinical symptoms being unspecific. Sometimes, SOX-10 positivity can mislead to malignant melanoma, as in our case. A broad panel of other immunohistochemical markers of melanocytic, epithelial, and mesenchymal differentiation, as well as the tumor proliferative index, are critical for the definitive diagnosis.

## Appendices

List of abbreviations

CD68 - cluster of differentiation 68 CDX2 - caudal-type homeobox 2 (CDX2) CK - cytokeratin CK7 - cytokeratin 7 CXPA - carcinoma ex-pleomorphic adenoma GFAP - glial fibrillary acidic protein HER-2 - human epidermal growth factor receptor 2 HMB-45 - human Melanoma Black 45 HU - Hounsfield units Ki-67 - marker of proliferation Kiel 67 Melan-A - melanoma antigen recognized by T-cells 1 p63 - tumor protein 63 panCK AE1/AE3 - aancytokeratin antibodies AE1 and AE3 PA - pleomorphic adenoma S100 protein - protein soluble in 100% ammonium sulfate at neutral pH SMA - smooth muscle actin SOX-10 - SRY-box transcription factor 10 TP53 - tumor protein p53
